# Endovascular therapy beyond 24 hours for anterior circulation large vessel occlusion or stenosis in acute ischemic stroke: a retrospective study

**DOI:** 10.3389/fneur.2023.1237661

**Published:** 2023-12-06

**Authors:** An Wen, Wen-feng Cao, Chao Zhao, Ling-feng Wu, Yong-liang Zhou, Zheng-bing Xiang, Wei Rao, Shi-min Liu

**Affiliations:** ^1^Department of Neurology, Jiangxi Provincial People's Hospital (The First Affiliated Hospital of Nanchang Medical College), Nanchang, Jiangxi, China; ^2^Department of Neurology, Xiangya Hospital, Central South University, Jiangxi Hospital, National Regional Center for Neurological Diseases, Nanchang, Jiangxi, China; ^3^Department of Neurology, Shangrao Municipal Hospital, Shangrao, Jiangxi, China

**Keywords:** acute ischemic stroke, delayed endovascular treatment, extended window, large vessel occlusion, magnetic resonance imaging

## Abstract

**Background:**

To assess the clinical and safety outcomes of endovascular treatment (EVT) administered more than 24 h after the onset of symptoms in patients with acute ischemic stroke resulting from anterior circulation large-vessel occlusion or stenosis (AIS-ACLVO/S).

**Methods:**

We enrolled consecutive AIS-ACLVO/S patients who received EVT in our hospital between January 2019 and February 2022 and divided them into two groups based on the time from AIS onset to EVT: EVT < 24 h group and EVT >24 h group. The successful reperfusion (modified thrombolysis in cerebral infarction, [mTICI] ≥2b), 90-day modified Rankin Scale score (mRS), intracranial hemorrhage (ICH), and symptomatic ICH (sICH), as well as mortality, were analyzed in the two groups of patients.

**Results:**

A total of 239 patients were included in the study, with 214 patients in the EVT < 24 h group (67.8 ± 0.8 years, 126 males) and 25 patients in the EVT > 24 h group (62.80 ± 2.0 years, 22 males). Both groups were similar in terms of hypertension, diabetes history, responsible vessels, and Alberta stroke program early computed tomography scores (*p* > 0.05). However, the EVT < 24 h group had significantly higher age, history of atrial fibrillation, proportion of patients receiving intravenous thrombolysis, and NIHSS scores before EVT than the EVT > 24 h group. AIS etiology differed between the groups, with more cases of large artery atherosclerosis in the EVT > 24-h group and more cases of cardioembolism in the EVT < 24-h group. Successful reperfusion (mTICI ≥2b), ICH, and sICH were similar between the groups. The 90-day functional independence rate (mRS ≤ 2) was significantly higher in the EVT > 24-h than in the EVT < 24-h group (80% vs. 39.7%, *p* < 0.001), while the 90-day mortality rate was lower in the EVT > 24-h group (0% vs. 24.8%, *p* < 0.001).

**Conclusion:**

In our study, we found that EVT beyond 24 h of symptom onset in patients selected with multimodal MR screening, was associated with high functional independence rates and low mortality. Larger or randomized studies are needed to confirm these findings.

## 1 Introduction

In 2015, results from five randomized controlled trials (RCT) established endovascular therapy (EVT) as the optimal treatment for acute ischemic stroke (AIS) involving large vessel occlusion (LVO), provided that it was performed within an effective time window. Specifically, the MR CLEAN, EXTEND-IA, and SWIFT PRIM trials were conducted within 6 h of stroke onset (1–3), while the REVASCAT trial was conducted within 8 h (4), and the ESCAPE trial within 12 h (5). In 2018, the DEFUSE 3 and DAWN trials extended the time window to 16–24 h to identify potentially salvageable tissues based on clinical symptoms and imaging mismatch (6, 7). However, in clinical practice, the onset time in patients is often unknown (> 24 h), or patients have mild early symptoms that later worsen, or patients are transferred from another hospital after 24 h. The optimal treatment strategy for these patients, i.e., medication or EVT, remains controversial.

The DEFUSE 3 study found that the ischemic penumbra persisted for more than 24 h in 18% of patients. If brain perfusion is not promptly restored, it could lead to worsening of clinical symptoms, resulting in poor outcomes and even mortality (8). The DAWN study revealed comparable outcomes in terms of successful revascularization, mRS ≤ 2 at 90 days, and symptomatic intracranial hemorrhage (sICH) between patients with AIS-LVO who met the inclusion criteria but received EVT more than 24 h from stroke onset and those who received EVT within 24 h (9), this suggests that the duration of the ischemic penumbra can extend beyond 24 h. However, both studies carefully selected patients for EVT by using RAPID software to assess infarct core and ischemic penumbra. Currently, in many countries and regions lacking access to such software, it remains unclear whether multimodal MRI can be used to assess and select patients for EVT, particularly in cases with symptom onset exceeding 24 h.

The objective of the study was to assess the clinical and safety outcomes of patients with AIS due to anterior circulation large-vessel occlusion or stenosis (ACLVO/S) who underwent screening with multimodal MRI and received Endovascular Thrombectomy (EVT) after 24 h from stroke onset, compared to patients treated within 24 h of stroke onset. We hypothesized that the outcomes of EVT beyond and before 24 h after stroke onset would be comparable.

## 2 Methods

We consecutively enrolled patients with AIS-ACLVO/S who were hospitalized at Jiangxi Provincial People’s Hospital between January 2019 and February 2022. Based on the duration from symptom onset to the implementation of EVT, patients were separated into two groups: EVT < 24 h group and EVT > 24 h group. All patients underwent non-contrast computed tomography (NCCT) to determine the Alberta stroke program early CT (ASPECT) score and exclude those with ICH. Patients who underwent EVT within 6 h also received magnetic resonance angiography (MRA) or computed tomography angiography (CTA) to evaluate the occluded or stenotic vessels, while patients who received EVT beyond 6 h after onset underwent diffusion-weighted imaging (DWI)/arterial spin labeling (ASL)/MRA to evaluate the occluded or stenotic vessels and DWI-ASPECT score (10).

Multimodal MR data acquisition: the acquisition sequence utilized readout segmentation of long variable echo-trains (RESOLVE) for DWI scans. The parameters for this axial scan were as follows: a repetition time (TR) of 5,600 ms, an echo time (TE) of 71 ms, a single average, a slice thickness of 5 mm, an interslice gap of 1.5 mm, a field of view (FOV) of 220 mm x 220 mm, an image matrix of 160 × 152, a diffusion value of b = 1,000 s/mm^2^, parallel acquisition using GRAPPAx2, and a total acquisition time of 1 min and 18 s. The ASL was performed in the axial orientation with the following parameters: TR of 4,600 ms, an TE of 16.16 ms, a single average, a slice thickness of 3 mm, an interslice gap of 1.5 mm, a FOV measuring 210 mm x 210 mm, an image matrix of 64 × 62.7, an inversion time (TI) of 2,510 ms, and a bandwidth of 2,694 Hz. The DWI/ASL data were visually assessed by experienced neurointerventional experts with over 20 years of experience. EVT was considered if the infarcted area did not cover more than one-third of the MCA blood supply territory and if there were patients with a penumbral region, at the joint discretion of the neurology and neurointerventional physicians, subject to approval by the patient’s family.

The eligibility criteria for EVT of AIS were as follows: (1) 18 years or older; (2) modified Rankin Scale (mRS) score ≤ 2 before AIS onset; (3) National Institutes of Health Stroke Scale (NIHSS) scores ≥6 before EVT; (4) ACLVO/S (>70%) involving the internal cerebral artery (ICA) and the first (M1) or second segment (M2) of the middle cerebral artery (MCA); (5) Patients only received IV thrombolysis inside the thrombolysis window. Patients were ineligible for EVT if they met any of the following criteria: (1) a recent history of major surgery within the month prior to AIS onset, (2) Uncontrolled hypertension defined as systolic blood pressure ≥ 185 mmHg or diastolic blood pressure ≥ 110 mmHg, (3) active bleeding or a predisposition to bleeding, (4) abnormal blood glucose levels below 2.7 mmol/L or above 22.2 mmol/L, and (5) severe cardiac, hepatic, or renal insufficiency, (6) received only arterial thrombolysis. Additionally, data were excluded from further analysis if the clinical information (time since symptom onset or last seen well, treatment with EVT, etc.) was incomplete or the patients declined to participate in the follow-up (missing data in any of the outcome measures).

Patient characteristics and clinical information, including sex, age, medical history, NIHSS scores prior to EVT, responsible vessels, ASPECT score (where DWI was used in the absence of CT), cause of AIS, time from stroke onset to puncture, time from stroke onset to exacerbation, time from exacerbation to puncture, time from puncture to reperfusion, postoperative modified thrombolysis in cerebral infarction (mTICI) classification, ICH, sICH (defined as imaging-confirmed ICH on imaging by NIHSS scores of ≥4 within 7 days of EVT) (11), 90-day mRS scores, and all-cause deaths within 90 days, were compared between both groups.

Patients underwent general anesthesia or local infiltrative anesthesia. After femoral artery puncture, an 8F sheath or a 6F long sheath was inserted. Subsequently, selective angiography was performed to identify the site of occlusion or stenosis. The EVT techniques used included direct aspiration, stent retriever, balloon dilation, and stent placement, and the operator decided on the best combination of these methods for each case. All devices utilized for EVT were granted approval by the China Food and Drug Administration (CFDA).

This study received approval from the Research Ethics Committee of Jiangxi Provincial People’s Hospital (2023–005) and was conducted in compliance with the principles of the Declaration of Helsinki. Written informed consent for the publication of their data was obtained from the patients or their authorized representatives.

### 2.1 Statistical analysis

Data analysis was conducted using SPSS statistical software (version 25.0; IBM, Armonk, USA). Visual and analytical methods, such as plots, histograms, and the Kolmogorov–Smirnov test, were employed to determine the normality of data distribution. Normally distributed variables were presented as mean ± standard deviation, and an independent samples t-test was employed to compare the two groups. Non-normally distributed variables were reported as median with interquartile range (M + IQR), and the Mann–Whitney U-test was employed to compare the two groups. Chi-square tests or Fisher’s exact tests were utilized to analyze the frequency (%) of categorical variables and qualitative data. Statistical significance was defined as a *p*-value less than 0.05.

## 3 Results

**Figure 1:**
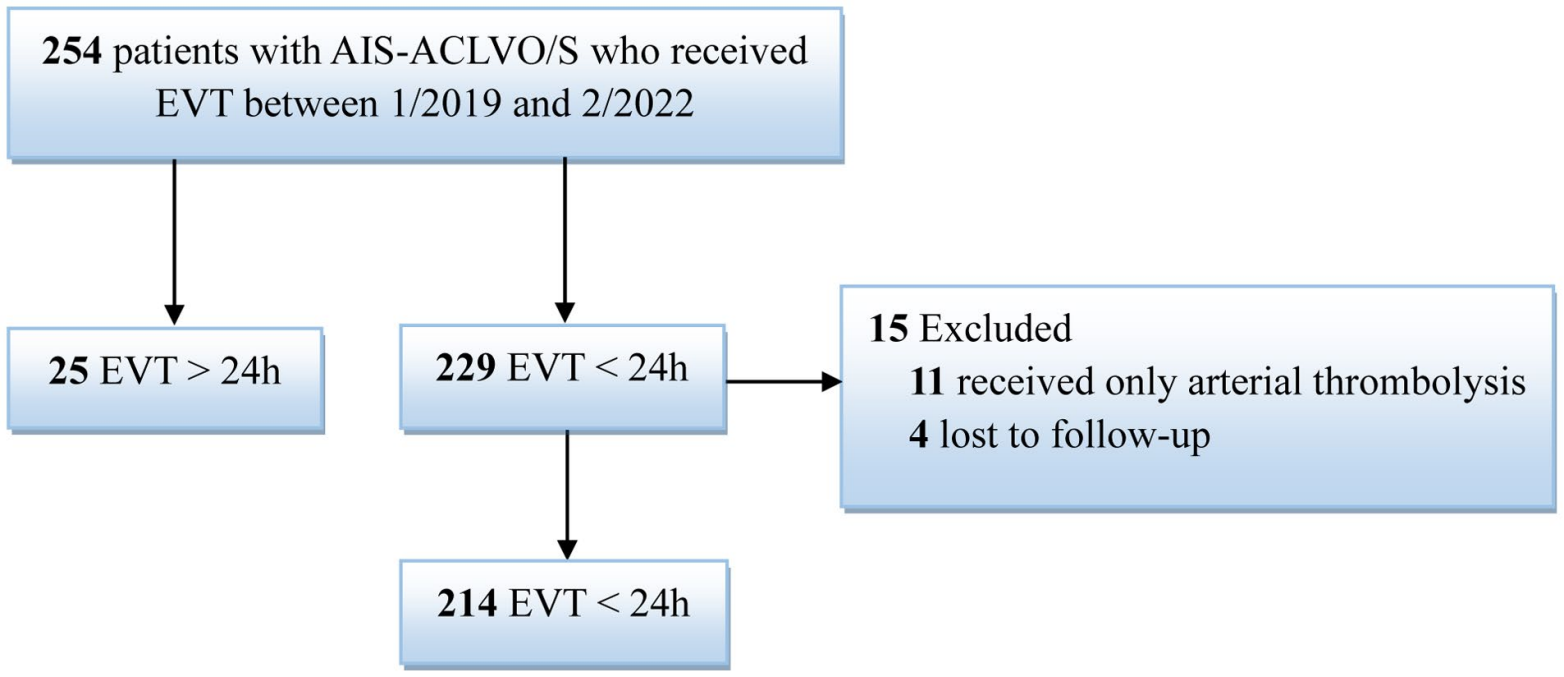
Flow chart of the study AIS-ACLVO/S: acute ischemic stroke resulting from anterior circulation large-vessel occlusion or stenosis. EVT: Endovascular treatment.

**Figure 2:**
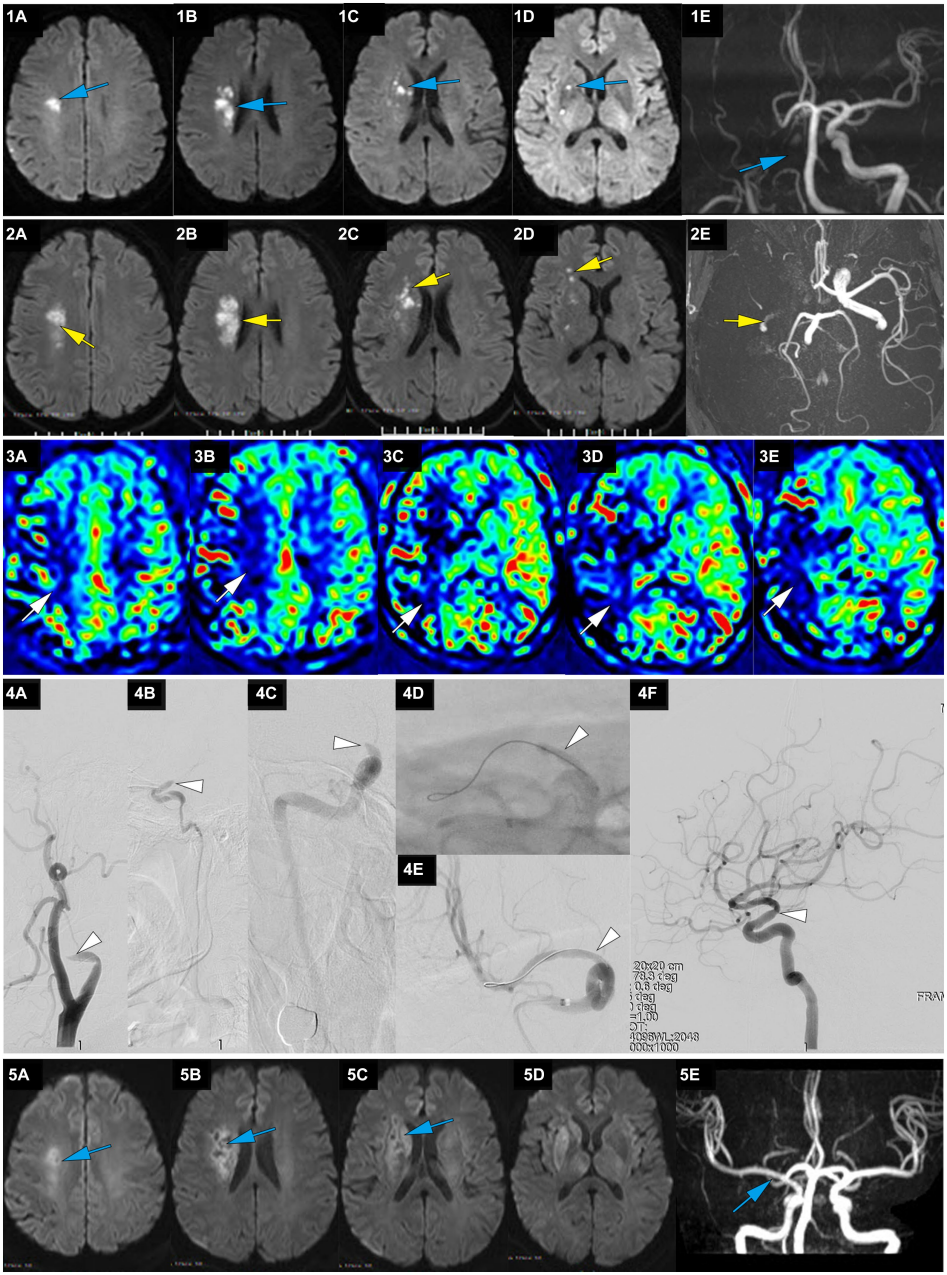
A representative case of AIS with occlusion of RMCA caused by large artery atherosclerosis. A 62-year-old male with a history of hypertension woke up with slurred speech and left-side numbness at 7:30 on May 25, 2021, and he was admitted to our hospital 14 h after the first AIS symptom of recognition (FAISSR). The NIHSS score upon admission was 3, and NCCT showed no signs of intracranial hemorrhage (ICH). The patient was treated with aspirin (100 mg/d) and clopidogrel (75 mg/d) dual antiplatelet therapy. On May 26, MRI-DWI revealed multiple scattered infarctions in the right corona radiata and basal ganglia (**1A-D**, blue), and MRA showed occlusion of the RICA (**1E**, blue). On May 27, the symptoms worsened to an NIHSS score of 8, and NCCT showed no ICH. The patient refused cerebral angiography and EVT, and Tirofiban was added to the treatment. On May 28, the patient’s symptoms worsened further to an NIHSS score of 15, MRI-DWI showed an increased infarction area (**2A-D**, yellow), MRA showed occlusion of the RICA (**2E**, yellow), and ASL indicated hypoperfusion in the right cerebral hemisphere (**3A-E**, white). The patient underwent emergency angiography 80 h and 30 min after FAISSR, which revealed slow blood flow in the RICA and occlusion of the proximal M1 segment (isolated MCA; **4A-C**, white). After balloon angioplasty, blood flow was restored to mTICI 3 grade (**4A-F**, white). The patient was discharged with an NIHSS score of 4. At the 3-month follow-up, the patient’s NIHSS score was 1, MRI-DWI showed gliosis in the old infarction area (**5A-D**, blue), and MRA indicated no restenosis in the RMCA (**5F**, blue).

**Figure 3:**
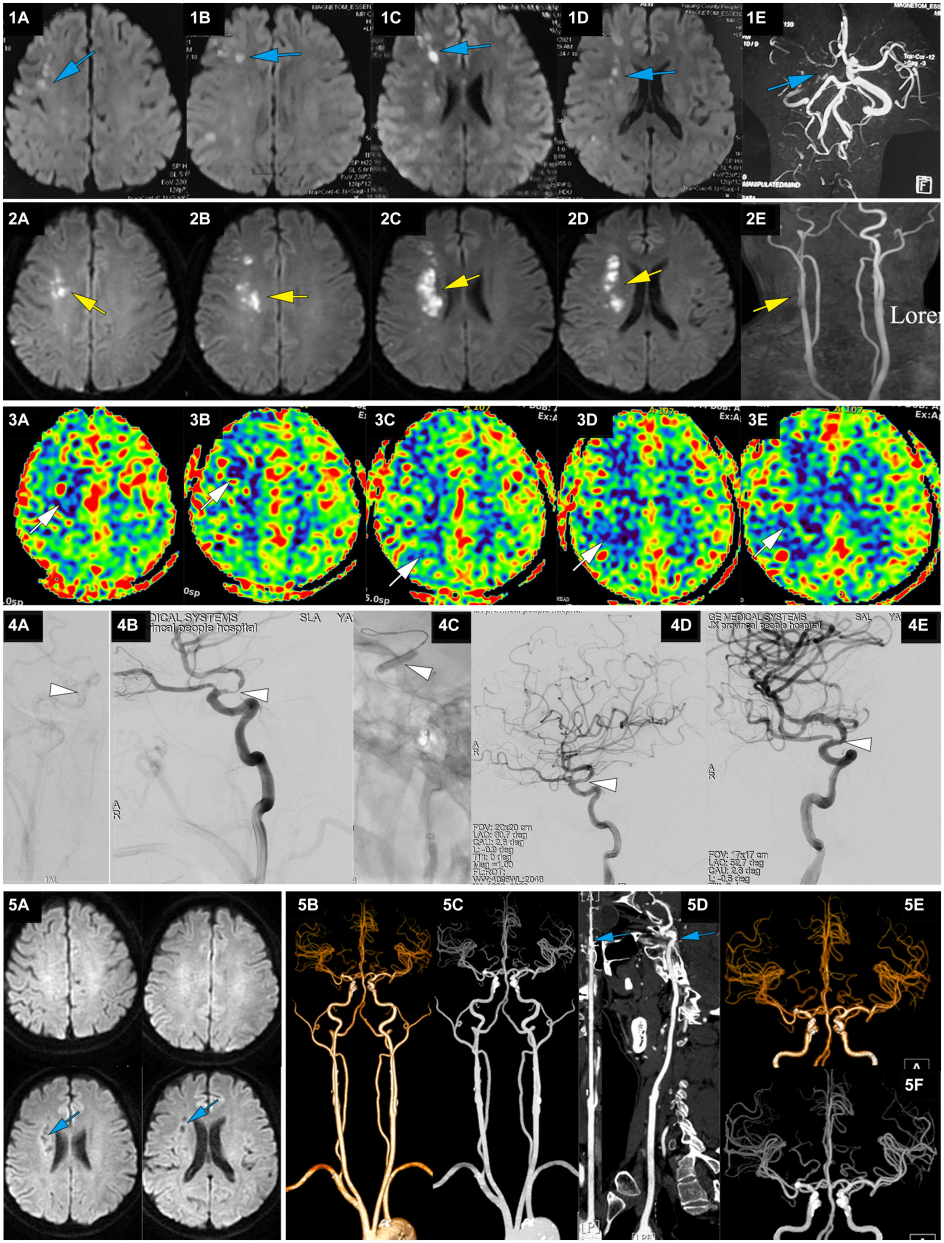
A representative case of AIS with RICA stenosis caused by large artery atherosclerosis. A 50-year-old male with a 5-year history of hypertension presented with sudden onset of left limb weakness at 13:00 on April 22, 2021. The main symptoms were a lack of flexibility in the left upper limb and slight weakness in the left lower limb, but he was able to walk independently. He was treated with dual antiplatelet therapy of aspirin and clopidogrel after CT showed no ICH. On April 23, MRI-DWI showed multiple scattered infarctions in the right cerebral hemisphere (**1A-D**, blue), and MRA suggested a possible occlusion of the RICA (**1E**, blue). At 7:00 on April 27, the patient’s condition worsened, with left upper limb weakness and difficulty walking. He was admitted to our hospital at 14:18 with an NIHSS score of 9, left upper limb muscle strength at grade 0, and lower limb muscle strength at grade 3. MRI-DWI showed an increased area of infarction (**2A-D**, yellow), MRA indicated that the RICA was slender (**2E**, yellow), and ASL showed hypoperfusion in the right cerebral hemisphere (**3A-E**, white). At 123.6 h after onset of AIS, emergency angiography showed severe stenosis in the terminal RICA (C7) and slow blood flow in the distal segment, with mTICI 2a grade (**4A,B**, white). Balloon dilation and stent placement (EZ 4.5*30 mm) were performed, and the stenosis was relieved with mTICI 3 grade (**4C-E**, white). Immediately after EVT, upper limb muscle strength recovered to the 3+ level, and the lower limb muscle strength recovered to level 4. At discharge, the NIHSS score was 1 point. At a 3-month follow-up after EVT, the NIHSS score was 0 points. MRI-DWI re-examination showed an old infarction (**5A**, blue), and CTA 1 year after EVT showed no stenosis in the RICA (**5B-F**, blue).

**Figure 4:**
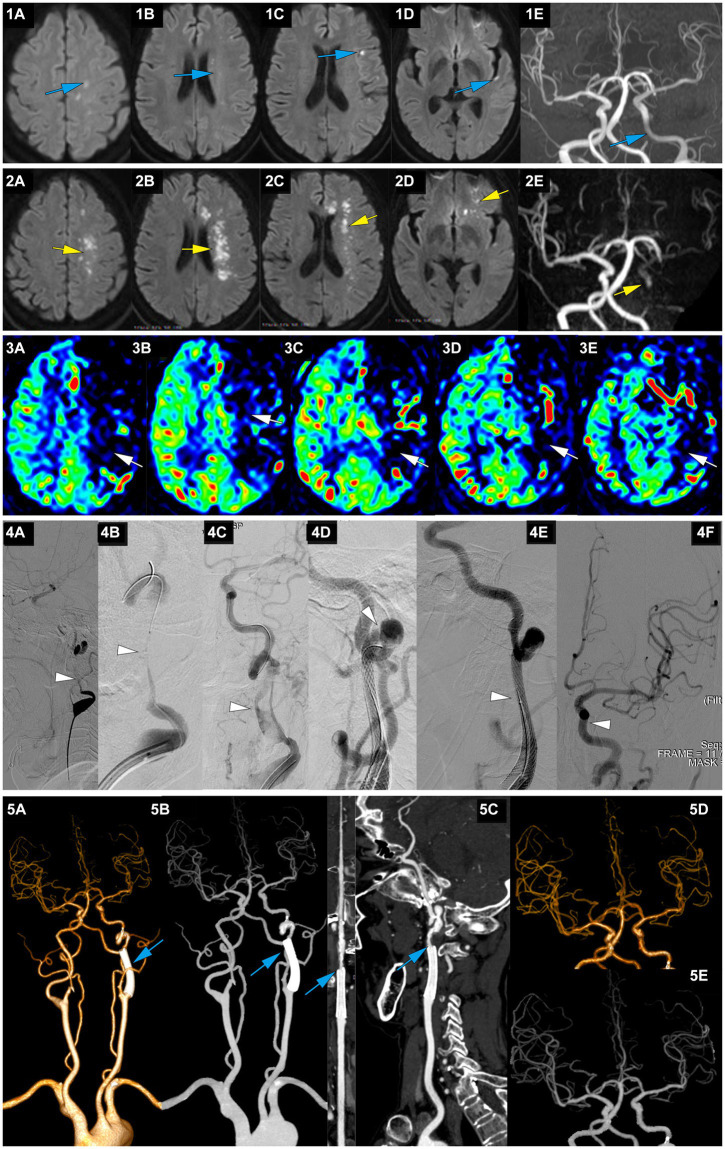
A representative case of AIS with occlusion of LICA caused by dissection. A 63-year-old female with no medical history experienced right limb weakness and slurred speech at 8 am on January 13, 2022. She was able to communicate and walk while holding objects normally (she carried a heavy load on her shoulder 2 days before the onset of symptoms). On January 15, MRI-DWI showed multiple punctate infarctions in the left hemisphere (**1A-D**, blue), and MRA indicated slightly diminished visualization of the LICA (**1E**, blue); she was treated with dual antiplatelet therapy of aspirin and clopidogrel. At 7:00 on January 21, her symptoms worsened, presenting as speechlessness and inability to move the right limbs. At 22:30 on January 21, the patient was transferred to our hospital. On admission, the NIHSS score was 12. MRI-DWI indicated a significant increase in the area of infarction (**2A-D**, yellow), MRA revealed occlusion of the LICA, with faint imaging of the distal MCA (**2E**, yellow), and ASL showed hypoperfusion in the left cerebral hemisphere (**3A-E**, white). At 207.3 h after the onset of AIS, emergency angiography showed LICA C1 segment occlusion with mTICI 1 grade (**4A**, white). A microguidewire and microcatheter were passed through the occlusion (**4B**, white), and after balloon dilation, a long segment dissection was found in the C1 segment (**4C**, white). A wallstent 7*40 mm was placed at the proximal end of the C1 segment curvature, but there was still a dissection in the distal end of the stent on angiography (**4D**, white). Therefore, a Solitaire 6*30 stent was bridged, and angiography showed that the stent completely covered the dissection (**4E**, white), with mTICI3 grade (**4F**, white). At discharge, the NIHSS score was 6. At 3 months postoperatively, the NIHSS score was 3, and the mRS score was 2; CTA showed no stenosis in the wallstent stent and mild stenosis at the Solitaire stent site (**5A-C**, blue), and intracranial vessels were normal (**5D-E**, blue).

In total, 254 consecutive patients with AIS-ACLVO/S who received EVT were included in this study. Eleven patients in the EVT < 24 h group who received only intra-arterial thrombolysis and four patients who were lost to follow-up were excluded, resulting in a final sample size of 239 patients (Table 1, Figure 1). The EVT < 24-h group consisted of 214 patients (126 males and 88 females) with an average age of 67.8 ± 0.8 years. The EVT > 24-h group included 25 patients (22 males and 3 females) with an average age of 62.8 ± 2.0 years. The reasons for delay of EVT beyond 24 h included transfer from another hospital (13 patients), worsening of symptoms during hospitalization (10 patients), and refusal to undergo surgery at disease onset (2 patients). In the EVT > 24 h group, 3 patients showed last-seen-well (LSW) at the beginning of the disease, and the symptoms of cerebral infarction gradually worsened during hospitalization. In addition, another 8 patients in this group had a sudden onset with mild symptoms at the beginning of the disease and developed LSW during hospitalization. In other words, in this group, 3 patients showed the onset form of LSW; Of the remaining 22 patients, some were able to provide detailed onset dates, while others had their initial onset observed by witnesses (see Figures 2–4).

**Table 1:** Demographic of the patients.

	EVT < 24 h (*N* = 214)	EVT > 24 h (*N* = 25)	Statistics	*p*
Age Age > 80, *n* (%)	67.8 ± 0.8 21(10)	62.80 ± 2.0 0	t = 2.143 χ^2^ = 1.605	0.033 0.205
Male, *n* (%)	126(58.9)	22(88)	χ^2^ = 8.051	0.005
Hypertension, *n* (%)	120(56.1)	13(52)	χ^2^ = 0.151	0.698
Diabetes mellitus, *n* (%)	25(11.7)	6(24)	χ^2^ = 2.017	0.156
Atrial fibrillation, *n* (%)	102(47.7)	4(16)	χ^2^ = 9.093	0.003
NIHSS before EVT	17.18 ± 0.38	12.8 ± 0.63	t = 3.867	0.000
ASPECTS(IQR)	7(6–8)	7(6–7)	z = −0.646	0.518
Occlusion/stenosis site, *n* (%)			2.054	0.339
Proximal ICA or tandem occlusion	55(25.7)	8(32)	χ^2^ = 0.458	0.499
Distal-ICA	21(9.8)	4(16)	χ^2^ = 0.374	0.541
MCA	138(64.5)	13(52)	χ^2^ = 1.50	0.221
Incomplete-occlusion, detected *n* (%)	-	4(16)	-	
Cause of AIS *n* (%)				
LAA	40(18.7)	16(64)	χ^2^ = 11.295	0.001
CE	98(45.8)	2(8)	χ^2^ = 13.140	0.000
AD	5(2.3)	7(28)	χ^2^ = 25.768	0.000
Undetermined	71(33.2)	0	χ^2^ = 11.800	0.001
Intravenous alteplase	99(46.3)	5(20)	χ^2^ = 6.281	0.012

NIHSS, National Institutes of Health Stroke Scale; EVT, Endovascular treatment; AIS, acute ischemic stroke; ASPECTS, Alberta stroke program early CT score; IQR, interquartile range; ICA, internal carotid artery; MCA, middle cerebral artery; LAA, large artery atherosclerosis; CE, Cardiac embolism; AD, artery dissection. *Aspiration or stent retriever + balloon dilatation and/or stenting.

As shown in Tables 1, 2, the percentage of patients aged >80 years, presence of related diseases, method of anesthesia, NIHSS scores before EVT, and ASPECT/DWI-ASPECT scores did not differ significantly between the two groups (*p* > 0.05). However, patients in the EVT > 24-h group were significantly younger (*p* < 0.05) and had a higher percentage of males than those in the EVT < 24-h group (*p* < 0.05). Additionally, atrial fibrillation incidence and intravenous thrombolysis were more prevalent in the EVT < 24-h group than in the EVT > 24-h group (*p* < 0.05).

**Table 2:** Compared clinical profile of the EVT < 24 h and EVT >24 h groups.

	EVT < 24 h (*N* = 214)	EVT > 24 h (*N* = 25)	Statistics	*p*
Type of EVT, *n* (%)				0.000
Aspiration	22(10.3)	0(0)	χ^2^ = 1.734	0.188
Stent retriever	152(71.0)	6(24)	χ^2^ = 22.096	0.000
Balloon dilatation and/or stenting	12(5.6)	14(56)	χ^2^ = 53.549	0.000
Combination*(A/S + B/S)	28(13.1)	5(20)	χ^2^ = 0.412	0.521
General anesthesia, n (%)	97(45.3)	8(32)	χ^2^ = 1.614	0.204
Workflow time (IQR)				
From AIS onset to puncture(min)	380 (283–500)	5,189 (3702–9,654)	z = −8.178	0.000
From AIS onset to exacerbation time (h)		81(52–140)		
From exacerbation to puncture time (min)		786 (588–1,089)		
From puncture to reperfusion time (min)	46(34–65)	65(44–105)	z = −2.624	0.009

EVT, Endovascular treatment; AIS, acute ischemic stroke; IQR, interquartile range; ICA, internal carotid artery; *Aspiration or stent retriever + balloon dilatation and/or stenting.

The median times from AIS onset to puncture and puncture to recanalization were shorter in the EVT < 24-h group than in the EVT > 24-h group (median time from AIS onset to puncture: 380 min [IQR: 283–500 min] vs. 5,189 min [IQR 3702–9,654 min]; median time from puncture to recanalization: 46 min [IQR: 34–65 min] vs. 65 min [IQR 44–105 min]) (*p* < 0.05; Table 2). The median times from AIS onset to exacerbation and exacerbation to puncture were 81 h and 786 min, respectively, in the EVT > 24-h group. There were no significant differences in successful reperfusion (mTICI ≥2b), ICH, or sICH between the two groups (*p* > 0.05). The EVT > 24-h group had a lower 90-day mortality rate (0% vs. 24.8%) and a higher percentage of 90-day mRS score ≤ 2 (80% vs. 39.7%) than did the EVT < 24-h group (*p* < 0.05). The median 90-day mRS score in the EVT > 24-h group was significantly lower than that in the EVT < 24-h group (1 [IQR: 1–2] vs. 3 [IQR: 1–5], *p* < 0.05; Table 3).

**Table 3:** Compared safety and efficacy of the EVT < 24 h and EVT >24 h groups.

	EVT < 24 h (*N* = 214)	EVT > 24 h (*N* = 25)	Statistics	*p*
mTICI≥2b *n* (%)	197(92.1)	24(96)	χ^2^ = 0.09	0.759
mRS at 90 days (IQR)	3 (1–5)	1 (1–2)	z = −3.537	0.000
mRS ≤2, *n* (%)	85 (39.7)	20 (80)	χ^2^ = 15.058	0.000
Safety outcomes *n* (%)				
ICH	76 (35.5)	6 (24)	χ^2^ = 1.317	0.251
sICH	16 (7.4)	1 (4)	χ^2^ = 0.052	0.819
Death	53 (24.8)	0	χ^2^ = 7.956	0.000

mTICI, modified thrombolysis in cerebral infarction; mRS = modified Rankin Scale; ICH, intracranial hemorrhage; sICH, symptomatic intracranial hemorrhage.

The etiologies of AIS differed between the EVT < 24 h and EVT > 24-h groups (*p* < 0.05; Table 2). Large-artery atherosclerosis (LAA) was more frequent in the EVT > 24-h group than in the EVT < 24-h group, whereas cardioembolism (CE) was more common in the EVT < 24-h group than in the EVT > 24-h group. Patients were further grouped according to their etiologies. Those with LAA+ artery dissection (AD) had lower pre-EVT NIHSS scores, 90-day mRS scores, ICH, and mortality rates than did those with CE (*p* < 0.05; Table 4), but there were no significant differences observed between the two groups in terms of (mTICI ≥2b) or sICH (*p* > 0.05; Table 4). Moreover, the LAA + AD group also had a higher percentage of 90-day mRS score ≤ 2 (*p* < 0.05; Table 4).

**Table 4:** Comparison of AIS due to LAA + AD and CE.

	LAA + AD (*N* = 68)	CE (*N* = 100)	Statistics	*p*
NIHSS before EVT	14.28 ± 0.56	17.87 ± 0.58	t = 4.241	0.000
mTICI≥2b n (%)	65 (95.6)	99 (99)	F	0.304
mRS at 90 days (IQR)	1.5 (0–4)	4 (1–6)	z = −3.713	0.000
mRS ≤ 2, *n* (%)	37(54.4)	36 (36)	χ^2^ = 5.584	0.018
Safety outcomes *n* (%)				
ICH	14 (20.1)	41(41)	χ^2^ = 7.658	0.006
sICH	2 (2.9)	8 (8)	χ^2^ = 1.057	0.304
Death	3 (4.4)	29(29)	χ^2^ = 15.870	0.000

LAA, large artery atherosclerosis; AD, artery dissection; CE, Cardiac embolism; NIHSS, National Institutes of Health Stroke Scale; mTICI, modified thrombolysis in cerebral infarction; mRS, modified Rankin Scale. ICH, intracranial hemorrhage; sICH, symptomatic intracranial hemorrhage.

## 4 Discussion

Currently, large-scale RCTs on EVT beyond 24 h after stroke onset are lacking, and the only published studies on this topic are retrospective studies or case reports. This study demonstrated that EVT beyond 24 h after symptom onset, using multi-modal MR guidance, had good safety and efficacy in AIS-ACLVO/S patients, with 80% of patients attaining functional independence (mRS ≤ 2) at 90 days. There was no increased risk of ICH or sICH in patients with EVT beyond 24 h after symptom onset compared with those with EVT within 24 h after stroke onset, and a lower mortality rate was noted among patients who underwent EVT beyond 24 h after the onset of symptoms.

A multinational retrospective observational cohort study (SELECT Late Study), which is currently the largest study on EVT beyond 24 h after stroke onset, compared the best medical management only (116 patients) with EVT (185 patients) in patients with AIS-ACLVO beyond 24 h of symptom onset and found that EVT was linked to improved functional independence but increased the risk of sICH (12). In addition, Purrucker et al. reviewed two local prospective recanalization databases from January 2014 to August 2021, including 43 patients who underwent EVT beyond 24 h (31 in the anterior circulation and 12 in the posterior circulation) and 2,304 patients who underwent EVT within 24 h (2048 in the anterior circulation and 256 in the posterior circulation). They found that the favorable outcomes (mRS ≤ 2) achieved with EVT beyond 24 h and within 24 h were 23.3 and 39.4%, respectively (*p* = 0.04), but no significant differences were observed between the two groups in terms of ICH or mortality (13). However, the two studies had no unified imaging inclusion criteria and had a long time span, resulting in significant differences in both imaging screening and surgical instrument methods. Moreover, British scholars reviewed EVT data from multiple centers in England, Wales, and Northern Ireland and compared two groups of patients, i.e., those with EVT conducted at 6–24 h (208 patients) and those with EVT conducted at >24 h (104 patients). There were no significant disparities observed in good functional outcomes (mRS ≤ 2), successful reperfusion, sICH, or mortality between the two groups (14).

Ha et al. (South Korean scholars) compared patients in three groups (i.e., EVT < 6 h, EVT 6–24 h, and EVT > 24 h) and found that CE was common in the EVT < 6 h group, whereas LAA was common in the EVT > 24-h group (15). In this study, the proportion of cases with LAA + AD in the EVT > 24-h group was as high as 92% (23/25), and the CE rate, the pre-EVT NIHSS score, ICH rate, and mortality rate were lower, while the 90-day favorable outcome (mRS ≤ 2) was higher in the LAA + AD group than in the CE group. Moreover, a meta-analysis of patients with AIS-LVO caused by LAA and CE treated with EVT revealed lower rates of successful reperfusion and functional independence, and a higher mortality rate in patients with LAA compared to those with CE (16). However, the direct intraarterial thrombectomy in order to revascularize acute ischemic stroke patients with large vessel occlusion efficiently in chinese tertiary hospitals(DIRECT-MT) study revealed a higher probability of patients with LAA attaining an mRS score of 0–1 than those with CE after EVT alone, and there was no statistically significant difference between the two groups in terms of death and sICH (17). These inconsistent findings suggested that diverse etiologies of AIS-ACLVO/S influence the results of EVT. The variability in causative factors across various time periods remain uncertain and thus warrant further investigation.

In this study, visual-only screening by DWI/ASL was used to enroll patients with stroke onset beyond 24 h. One previous study employed multimodal MR for patient screening and demonstrated that DWI/PWI alone could effectively identify patients with AIS-LVO for timely EVT (18). Additionally, DWI-FLAIR mismatch has demonstrated safety and efficacy in the screening of patients with stroke of unclear onset (19). However, this approach is largely based on visual assessments by experienced neurointerventionists or neuroimaging specialists, and had no established quantitative criteria, which might lead to a selection bias. In addition, patient enrollment in the DEFUSE 3 and DAWN studies involved rigorous imaging screening and required the use of advanced software systems to assess the ischemic penumbra, which may not be available at all medical centers and hospitals, thus potentially denying the opportunity of EVT for some patients. Notably, the MR CLEAN-LATE study found that it is safe and effective to perform EVT for patients with ACLVO in the late window (6–24 h from symptom onset or last seen well) when selecting patients based exclusively on the presence of collateral flow identified through CTA screening (20).

In the EVT > 24 h group of this study, the average time from symptom onset to puncture was 86.5 h, with the longest duration observed in one patient being 9.5 days. While “Time is brain” has traditionally determined cerebral infarction outcomes, recent research suggests that the quality of collateral circulation in stroke patients may have a greater impact on infarct size and clinical outcomes than time alone (21). The rate of infarct core expansion into the penumbral region is primarily influenced by collateral circulation (22). Additionally, the conventional “time window” concept is gradually shifting toward a “tissue window” perspective (23). The DAWN study found that the good collaterals patients exhibited milder symptoms at the time of onset, smaller infarct volumes, less infarct progression at 24 h, and a higher rate of favorable outcomes at 90 days compared to the poor collaterals patients (24). Among patients meeting the inclusion criteria of the DAWN study, the maximum time for EVT was 6 days. This also suggests that the duration of the ischemic penumbra may be longer in some patients than previously anticipated (9). A retrospective study revealed that only about 25% of ACLVO patients had an infarct core >70 mL within the first 6 h of symptom onset. Within the initial 24 h, 55% had an infarct core <30 mL and 43% (80 out of 185) of patients had an infarct core of ≤10 mL. These findings indicate that patients with poorer collateral circulation tend to progress rapidly, whereas more than half of the patients experience a slower infarct progression (25).

Two interesting phenomena were observed in this study. First, the EVT > 24-h group had significantly younger patients compared to the EVT < 24-h group, which corresponds to the results of a study conducted by Ha et al. (15). Second, the preoperative NIHSS score was lower in the EVT > 24-h group than in the EVT < 24-h group, indicating that the former patients was less symptomatic; the result aligns with the findings of two other studies (14, 15). This finding might have contributed to the favorable clinical outcomes observed in the EVT > 24-h group as compared with the EVT < 24-h group.

It is possible that surgeons tended to select younger patients with milder clinical symptoms for EVT, in contrast, medical therapy was more commonly administered to older patients with more severe symptoms. The baseline characteristic imbalances combined with the counterintuitive better outcomes in the EVT >24 h compared to the EVT <24 h group indicate a (strong) presence of selection bias. However, despite this bias, our findings do indicate that with proper selection (multimodal MR), it may be possible to identify a group of patients in the >24 h window that will benefit from EVT.

This study was a retrospective study, with visual screening of patients, with potential selection bias and a small sample size, Thus, whether these confounders led to the present findings remains to be clarified in large-scale RCT. Currently, the Large Artery occlusion Treated in Extended time with Mechanical Thrombectomy (LATE-MT, NCT05326932) RCT is recruiting patients with a proposed extension of the EVT time window to 72-h post-onset.

## 5 Conclusion

In our study, we found that EVT beyond 24 h of symptom onset in patients selected with multimodal MR screening, was associated with high functional independence rates and low mortality. Larger or randomized studies are needed to confirm these findings.

## Data availability statement

The original contributions presented in the study are included in the article/Supplementary material, further inquiries can be directed to the corresponding author.

## Ethics statement

The studies involving humans were approved by Jiangxi Provincial People’s Hospital (The First Affiliated Hospital of Nanchang Medical College). The studies were conducted in accordance with the local legislation and institutional requirements. The ethics committee/institutional review board waived the requirement of written informed consent for participation from the participants or the participants’ legal guardians/next of kin.

## Author contributions

S-mL: designed the study and conceived of the manuscript, drafted, and provided critical revisions. AW and W-fC: did the literature review and wrote first draft. CZ, L-fW, Y-lZ, Z-bX, and WR are surgical operators. All authors contributed to the article and approved the submitted version.
